# Topographical anatomy of the septum verum and its white matter connections

**DOI:** 10.1038/s41598-024-68464-x

**Published:** 2024-08-05

**Authors:** Laszlo Barany, Cintia Meszaros, Alan Alpar, Oliver Ganslandt, Nirjhar Hore, Daniel Delev, Oliver Schnell, Peter Kurucz

**Affiliations:** 1 Department of Neurosurgery, Universitätsklinikum Erlangen, Friedrich-Alexander-Universität Erlangen-Nürnberg (FAU), Erlangen, Germany; 2 Department of Anatomy, Semmelweis University, Budapest, Hungary; 3 SE NAP Research Group of Experimental Neuroanatomy and Developmental Biology, Semmelweis University, Budapest, Hungary; 4 Department of Neurosurgery, Katharinenhospital, Klinikum Stuttgart, Stuttgart, Germany

**Keywords:** Anatomy, Medical research

## Abstract

The human septum verum represents a small but clinically important region of the brain. Based on the results of animal experiments, the stimulation of its medial part was recently proposed with various indications like epilepsy or cognitive impairment after traumatic brain injury. The aim of our study was to present the anatomical relationships of the human septum verum using fiber dissection and histological analysis to support its research and provide essential information for future deep brain stimulation therapies. 16 human cadaveric brains were dissected according to Klingler’s method. To validate our macroscopical findings, 12 samples obtained from the dissected brains and 2 additional specimens from unfrozen brains were prepared for histological examinations. We identified the following white matter connections of the septum verum: (1) the precommissural fibers of the fornix; (2) the inferior fascicle of the septum pellucidum; (3) the cingulum; (4) the medial olfactory stria; (5) the ventral amygdalofugal pathway; (6) the stria medullaris of the thalamus and (7) the stria terminalis. Moreover, we could distinguish a less-known fiber bundle connecting the postcommissural column of the fornix to the stria medullaris of the thalamus and the anterior thalamic nuclei. In this study we present valuable anatomical information about this region to promote safe and effective deep brain stimulation therapies in the future.

## Introduction

The septum telencephali is a midline forebrain region between the cerebral hemispheres. It was considered as a vestigial structure for a long time without any attributed function. Andy and Stephan showed first in their comparative histological study that this region is actually well-developed also in humans^1^. They distinguished two parts of the human septum, the dorsal located septum pellucidum and the subjacent laying septum verum that contains the septal nuclei and corresponds to the septum of the animals^1^.

The exact functions of the septum are not fully understood in humans, however its numerous connections to other brain regions reflect its role in complex operations. The hippocampal oscillation is essential for various behavioral, cognitive and memory functions. The medial part of the septum plays an important role in its generation^2^, the alteration or disturbance of which is a common observation in various pathological conditions. Animal experiments proved the therapeutic effect of its stimulation by restoring or imitating the physiological oscillation^2^. Based on these promising results, the medial septum was proposed for a possible target of deep brain stimulation (DBS) with the indications of treatment-resistant epilepsy^3^, cognitive impairment after traumatic brain injury^4^, Alzheimer’s disease^2^, various psychiatric disorders^2^ and chronic pain^2^.

Safe and effective DBS needs the complex spatial understanding of the underlying anatomy. Although some white matter connections related to the septal area have already been examined by fiber dissection^5–10^, no study yet focused on the anatomical relationships of the septum verum using this method so far. Therefore, the aim of our study was to provide a topographical description of the human septum verum and its main white matter connections using fiber dissection technique supported with histological validation.

## Methods

16 human cadaveric brains (32 hemispheres) were involved in this study.

### Fiber dissections

14 brains were prepared using a modified method of Klingler^11^. The precise separation of the white matter fibers was achieved using a special fixation method of the cadaveric brains. The first step was the cannulation of the internal carotid and vertebral arteries of the cadaveric heads, then the blood vessels supplying the brain were flushed with 0.9% saline and perfused with 4% formalin solution. This in-situ pre-fixation prevented the deformation of the brains which is crucial to preserve the original topographical position of the anatomical structures. After a maximum of 72 h post-mortem time, the brains were carefully removed from the skull and immersed in 4% formalin solution as usual for at least 2 months. After fixation, the brains were frozen in water at − 30 °C for 2 weeks. Subsequently, the brains were thawed and washed out, cut in the mid-sagittal plane and stored in 4% formalin solution between the dissection steps. The dissections were carried out in a medial to lateral direction under the magnification of a stereomicroscope (Wild Heerbrugg, Switzerland) using neurosurgical dissectors, micro-forcipes, and micro-scissors. Photos were taken in every step of the dissections using 50- and 100-mm macro-objectives mounted on a Canon EOS 5D Mark II body (Canon Inc., Japan).

### Histology

Histological analyses were performed to validate the accuracy of the fiber orientations obtained with macroscopical dissections. A tissue block containing the area between the anterior commissure and the mamillary bodies was removed from two formalin-fixed but not frozen human brains to perform serial sections in the frontal and horizontal planes. During the fiber dissections, 12 additional samples were removed randomly from the investigated areas to validate the course of the dissected fibers. All histological samples were embedded in paraffin then cut in 8 µm thick slices and stained with neurofibril silver impregnation according to Krutsay^12^ or Luxol fast blue combined with Sirius red. While silver impregnation of neurofibrils stains the cytoskeletal components of neurons making also unmyelinated fibers visible, Luxol fast blue with Sirius red shows the myelinated fibers in green and the blood vessels in red colour due to the presence of collagen fibers in their walls, allowing the differentiation of these structures.

### Ethical statement

The cadavers presented in this study were donated with educational and research purposes to the Department of Anatomy, Histology and Embryology of the Semmelweis University, Budapest, Hungary with a written informed consent during their lifetime. Body donation and the use of cadavers are permitted and controlled by Section 222 of Act No. CLIV of 1997 on health as well as the “Regulations on the handling of cadavers donated for educational and research purposes” of the Semmelweis University [Decision No. 65/2022. (IX.02.) of the Semmelweis University Senate]. As the methods are routinely used in the medical education, an approval by an institutional committee was not necessary. The methods as well as the handling of cadavers were performed in accordance with the above-mentioned guidelines.

### Conference presentation

Parts of this manuscript were previously presented as ePoster at the 74. Annual Meeting of the German Society of Neurosurgery (DGNC) in 2023.

## Results

### Macroscopic results

**Figure 1:**
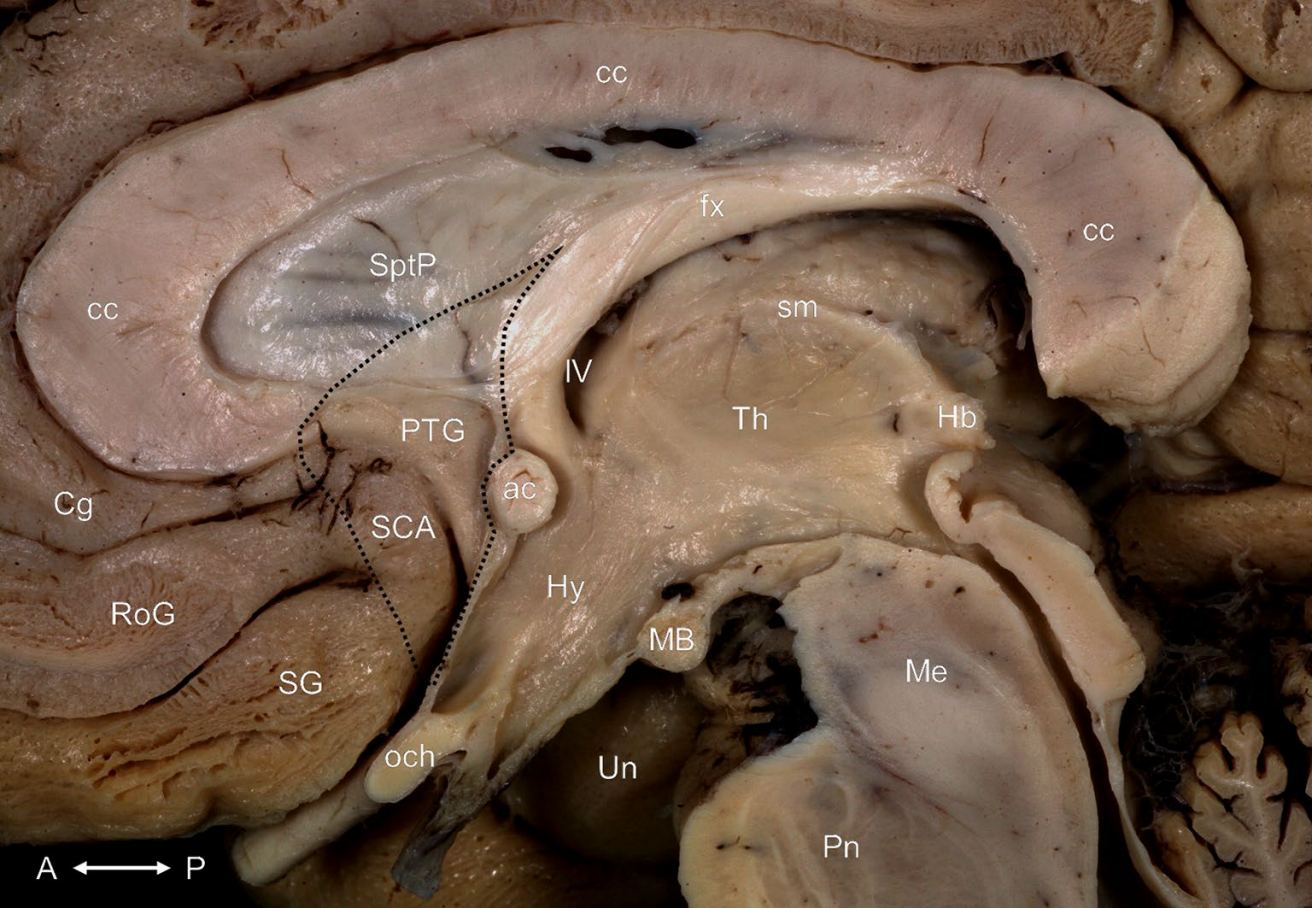
Overview of the medial surface of the brain before dissection. The septum verum is indicated with a dotted line. ac: anterior commissure; cc: corpus callosum; Cg: cingulate gyrus; fx: fornix; Hb: habenula; Hy: hypothalamus; IV: interventricular foramen; MB: mamillary body; Me: mesencephalon; och: optic chiasma; Pn: pons; PTG: paraterminal gyrus; RoG: rostral gyrus; SCA: subcallosal area; SG: straight gyrus; sm: stria medullaris of thalamus; SptP: septum pellucidum; Th: thalamus; Un: uncus. Compass: A: anterior; P: posterior.

The septum verum was identified as a nearly triangular grey matter area on the medial surface of the brain, composing the inferior part of the medial wall of the lateral ventricle’s frontal horn and containing the ventral part of the septum pellucidum, the paraterminal gyrus (also known as the subcallosal gyrus) and the subcallosal area (also known as the parolfactory area). It was bordered dorsally by the septum pellucidum and rostrally by the rostrum of the corpus callosum as well as the cingulate gyrus, the rostral gyrus and the gyrus rectus, from which it was separated by the poorly defined anterior parolfactory sulcus. Its posterior border was composed by the column of the fornix, the anterior commissure and the lamina terminalis (Fig. 1). The dorsal part of its lateral surface was covered by ventricular ependyma, while its ventral part was laterally continuous with the grey matter of the accumbens nucleus and the bed nucleus of the stria terminalis.

Our fiber dissections revealed the following white matter connections of the septum verum: (1) the precommissural fibers of the fornix; (2) the inferior fascicle of the septum pellucidum; (3) the cingulum; (4) the medial olfactory stria; (5) the ventral amygdalofugal pathway; (6) the stria medullaris of the thalamus and (7) the stria terminalis.

(1) The precommissural fibers of the fornix

**Figure 2:**
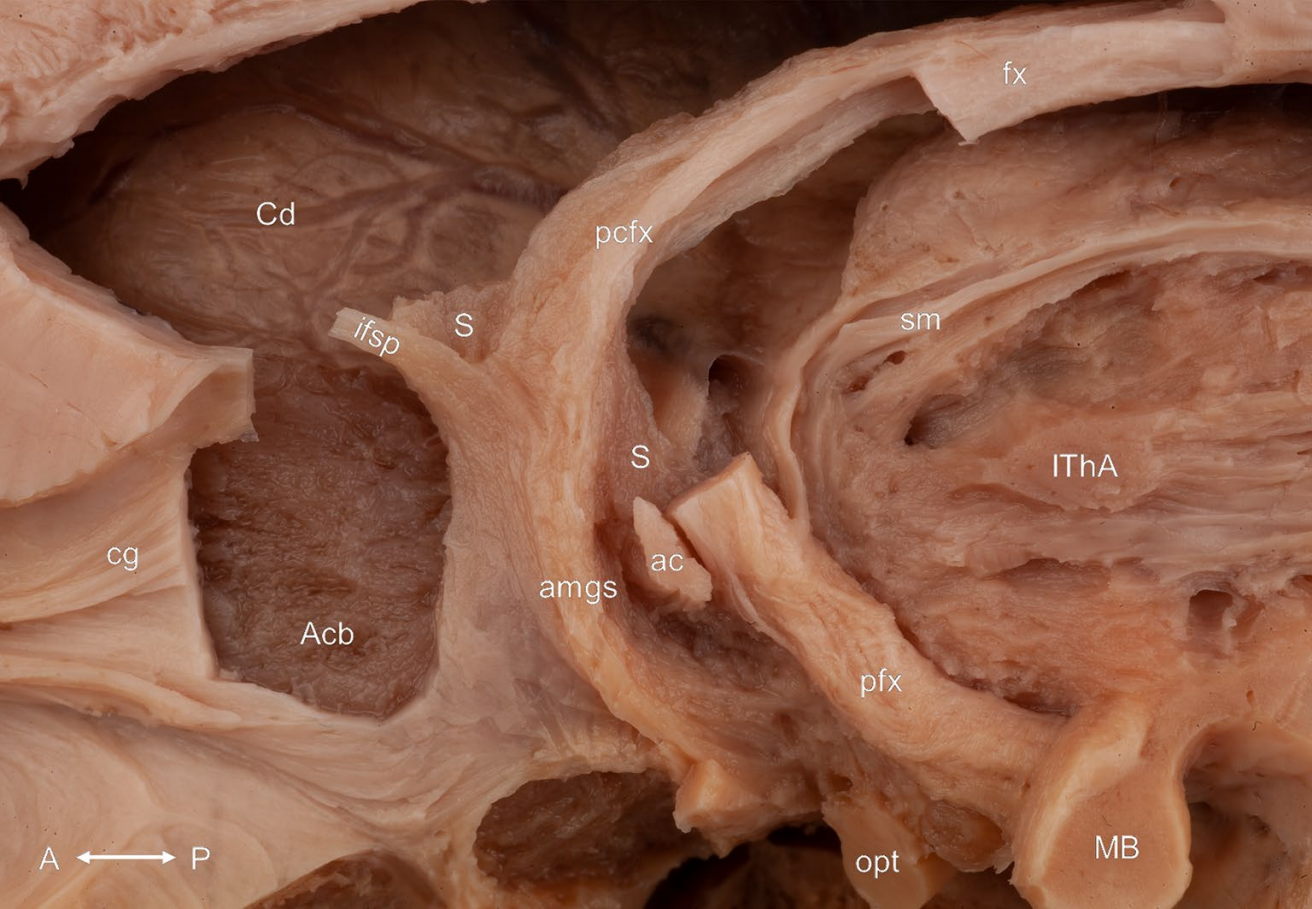
White matter connections of the septal region from medial after the removal of the septum pellucidum and most of the septal nuclei. Note the continuity between the septum verum, the caudate nucleus and the accumbens nucleus. Acb: accumbens nucleus; ac: anterior commissure; amgs: amygdaloseptal fibers; Cd: caudate nucleus; cg: cingulum bundle; fx: fornix; ifsp: inferior fascicle of the septum pellucidum; IThA: interthalamic adhesion; MB: mamillary body; opt: optic tract; pcfx: precommissural fornix; pfx: postcommissural fornix; S: septal nuclei; sm: stria medullaris of the thalamus. Compass: A: anterior; P: posterior.

**Figure 3:**
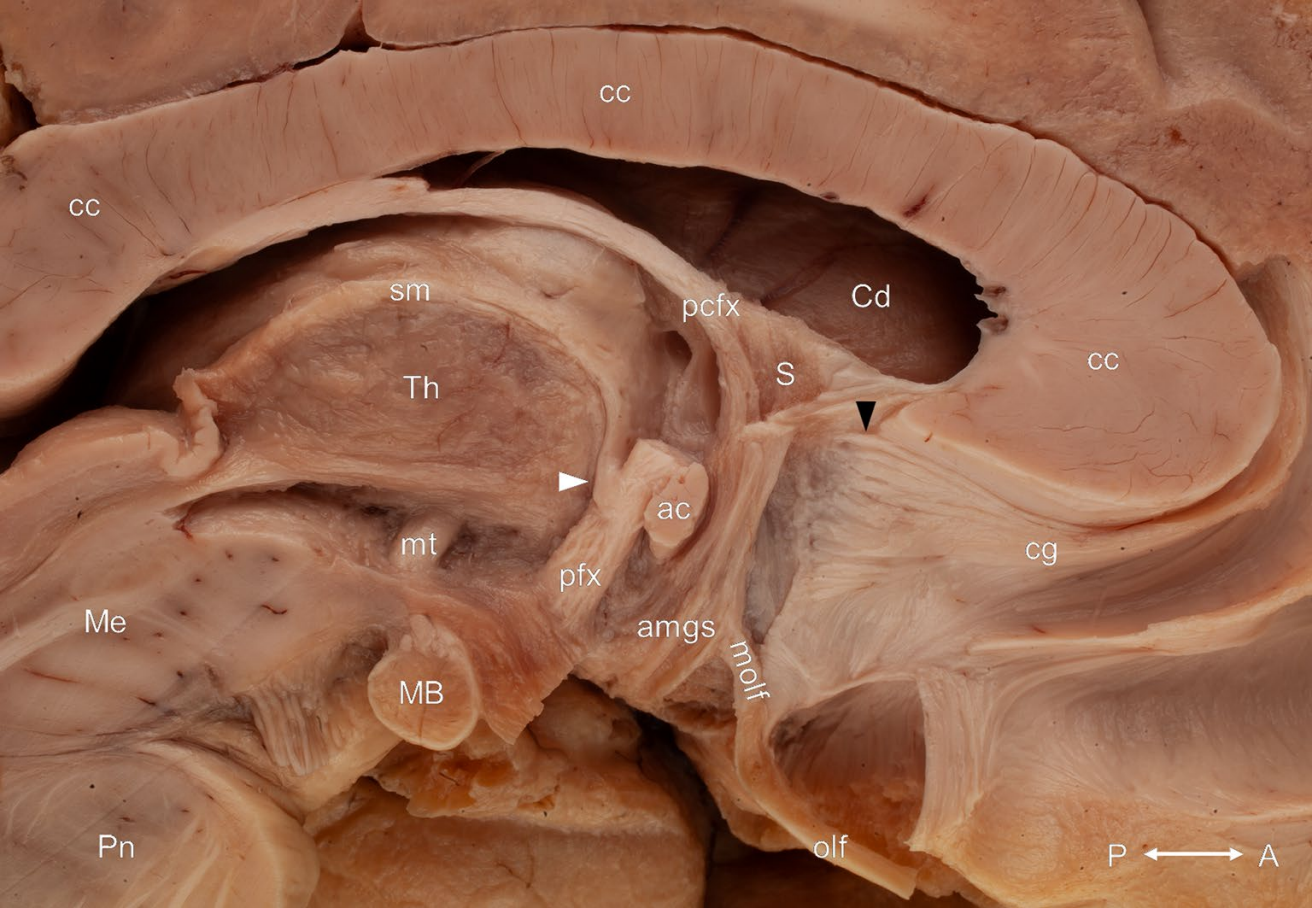
White matter connections of the septum verum from medial after the removal of the septum pellucidum, the grey matter of the subcallosal area and the diagonal band of Broca. The postcommissural fibers of the body of the fornix have been partially removed. The black arrowhead indicates a fiber bundle belonging to the cingulum and originating in the rostral part of the septum verum. This bundle was covered medially by the diagonal band of Broca. The connection between the postcommissural column of the fornix and the stria medullaris of the thalamus is marked with white arrowhead. ac: anterior commissure; amgs: amygdaloseptal fibers; cc: corpus callosum; Cd: caudate nucleus; cg: cingulum bundle; MB: mamillary body; Me: mesencephalon; molf: medial olfactory stria; mt: mamillothalamic tract; olf: olfactory tract; Pn: pons; pcfx: precommissural fornix; pfx: postcommissural fornix; S: septal nuclei; sm: stria medullaris of the thalamus; Th: thalamus. Compass: A: anterior; P: posterior.

The precommissural fornix was the first revealed connection of the septum verum. These fibers coursed in the lateral part of the corpus of the fornix and left its main bundle just above the interventricular foramen. After a short course in anteroinferior direction, they terminated in the septal nuclei rostral to the anterior commissure. A thin grey matter substance corresponding to the most caudal portion of the septal nuclei was identifiable on the dorsal side of these fibers until the interventricular foramen (Figs. 2, 3, 5).

(2) The inferior fascicle of the septum pellucidum

This pathway originated from the septal nuclei and its fibers intermingled with the terminating fibers of the precommissural fornix. After a straight course in anterosuperior direction it joined the fasciculus superior of the septum pellucidum at the genu of the corpus callosum then turned lateral and were lost between the commissural fibers of the corpus callosum (Figs. 2, 5, 6).

(3) The cingulum

The cingulum was identified as a prominent white matter tract coursing parallel to the corpus callosum along its dorsal surface. Ventral to the genu of the corpus callosum, a fiber bundle belonging to the cingulum was revealed originating in the rostral part of the septum verum, just ventral to the fibers of the inferior fascicle of the septum pellucidum. It was covered medially by the most superficial part of the vertical limb of the diagonal band of Broca that intermingled with this bundle (Figs. 2, 3, 6).

(4) The medial olfactory stria

As the olfactory tract reached the anterior perforated substance, the medial olfactory stria left it and coursed in a dorsomedial direction towards the septal area. The terminating fibers of this bundle were found in the rostral part of the septum verum just posterior to the fibers of the cingulum (Figs. 3, 6).

(5) The ventral amygdalofugal pathway

**Figure 4:**
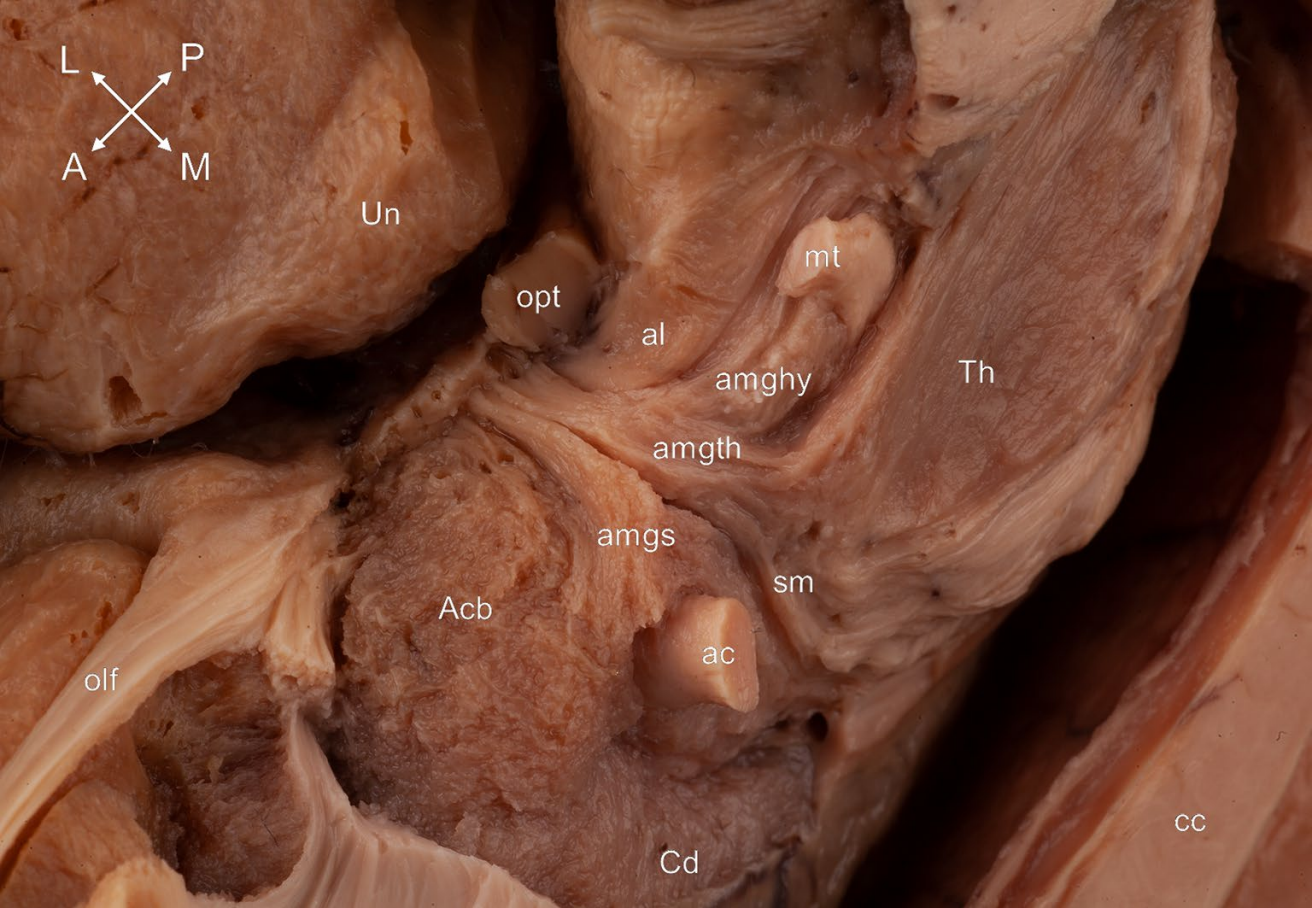
Topography of the connections coursing in the region of the basal forebrain from a mediobasal point of view. The amygdaloseptal fibers run as part of the diagonal band of Broca to the septal nuclei, just anterior to the amygdalothalamic fibers. Acb: accumbens nucleus; ac: anterior commissure; al: ansa lenticularis; amghy: amygdalohypothalamic fibers; amgs: amygdaloseptal fibers; amgth: amygdalothalamic fibers; cc: corpus callosum; Cd: caudate nucleus; mt: mamillothalamic tract; olf: olfactory tract; opt: optic tract; sm: stria medullaris of the thalamus; Th: thalamus; Un: uncus. Compass: A: anterior; L: lateral; M: medial; P: posterior.

The amygdaloseptal fibers connecting the ipsilateral amygdala to the septal nuclei were revealed after the removing of the grey matter of the diagonal band of Broca. This pathway consisted of a horizontal and a vertical limb. The horizontal one was located on the basal surface of the brain and ran parallel but more superficially to the anterior commissure. The inferior thalamic peduncle containing the amygdalothalamic and amygdalohypothalamic fibers coursed directly caudal to it. The vertical part of the amygdaloseptal fibers coursed on the medial surface of the brain in the area of the paraterminal gyrus and a part of this bundle joined the subgenual cingulum (Figs. 2, 3, 4).

(6) The stria medullaris of the thalamus

**Figure 5:**
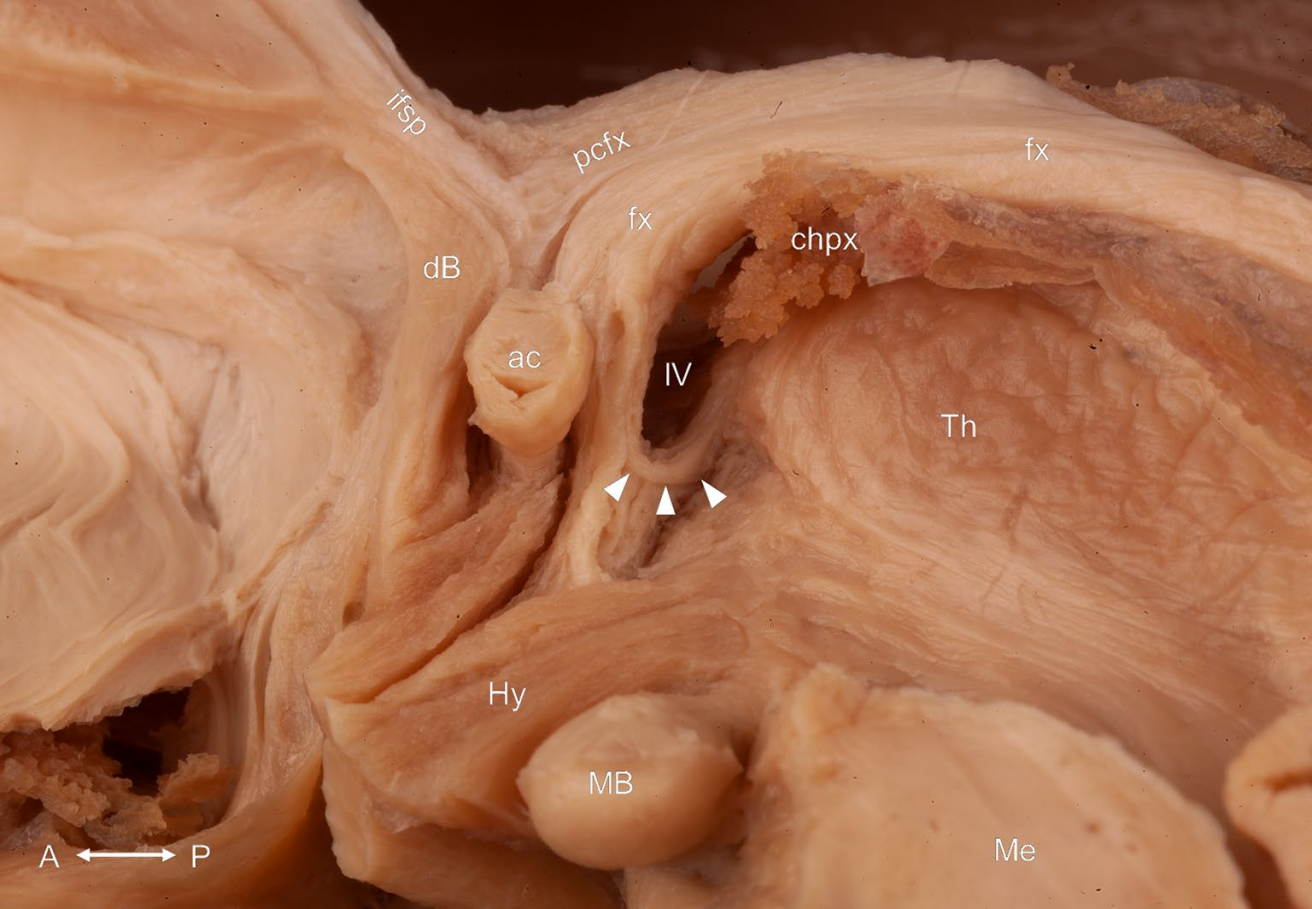
Topographical relationships of the septum verum from medial. The hairpin-like course of the fiber bundle connecting the postcommissural column of the fornix with the stria medullaris of the thalamus and the anterior thalamic nuclei is marked with white arrowheads at the inferior border of the interventricular foramen. ac: anterior commissure; chpx: choroid plexus; dB: diagonal band of Broca; fx: fornix; Hy: hypothalamus; ifsp: inferior fascicle of the septum pellucidum; IV: interventricular foramen; MB: mamillary body; Me: mesencephalon; pcfx: precommissural fornix; Th: thalamus. Compass: A: anterior; P: posterior.

**Figure 6:**
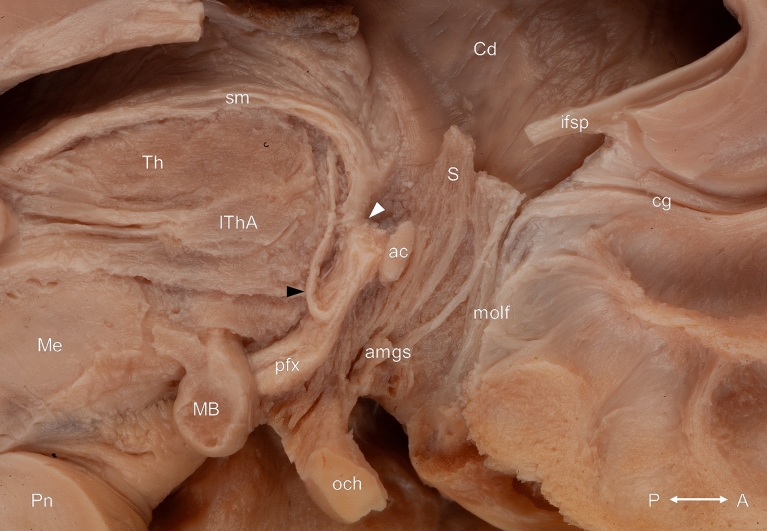
The cautious removal of the ependymal layer of the medial surface of the thalamus revealed the fiber bundle connecting the postcommissural column of the fornix to the anterior thalamic nuclei (black arrowhead), just distal to its connection with the stria medullaris of the thalamus (white arrowhead). ac: anterior commissure; amgs: amygdaloseptal fibers; Cd: caudate nucleus; cg: cingulum bundle; ifsp: inferior fascicle of the septum pellucidum; IThA: interthalamic adhesion; MB: mamillary body; Me: mesencephalon; molf: medial olfactory stria; och: optic chiasma; Pn: pons; pfx: postcommissural fornix; S: septal nuclei; sm: stria medullaris of the thalamus; Th: thalamus. Compass: A: anterior; P: posterior.

The stria medullaris of the thalamus was mainly composed of fibers coursing from the posterior border of the interventricular foramen to the ipsilateral habenula at the junction of the medial and dorsal surface of the thalamus. However, fibers crossing the midline through the habenular commissure and the interthalamic adhesion in its most superficial part as well as fibers between the anterior thalamic nuclei and the habenula (thalamohabenular fibers) in its deepest part were present in all cases. Fibers leaving the postcommissural column of the fornix at the level of the anterior commissure were also constant findings during our dissections. After a short hairpin-like course along the inferior border of the interventricular foramen, these fibers joined the stria medullaris of the thalamus or ran towards the anterior nuclei of the ipsilateral thalamus. They formed either a single bundle or the ones coursing to the thalamus left the postcommissural fornix a few millimeters distal to the fibers connecting it to the stria medullaris of the thalamus (Figs. 2, 3, 4, 5, 6, 8, 9).

(7) The stria terminalis (dorsal amygdalofugal pathway)

**Figure 7:**
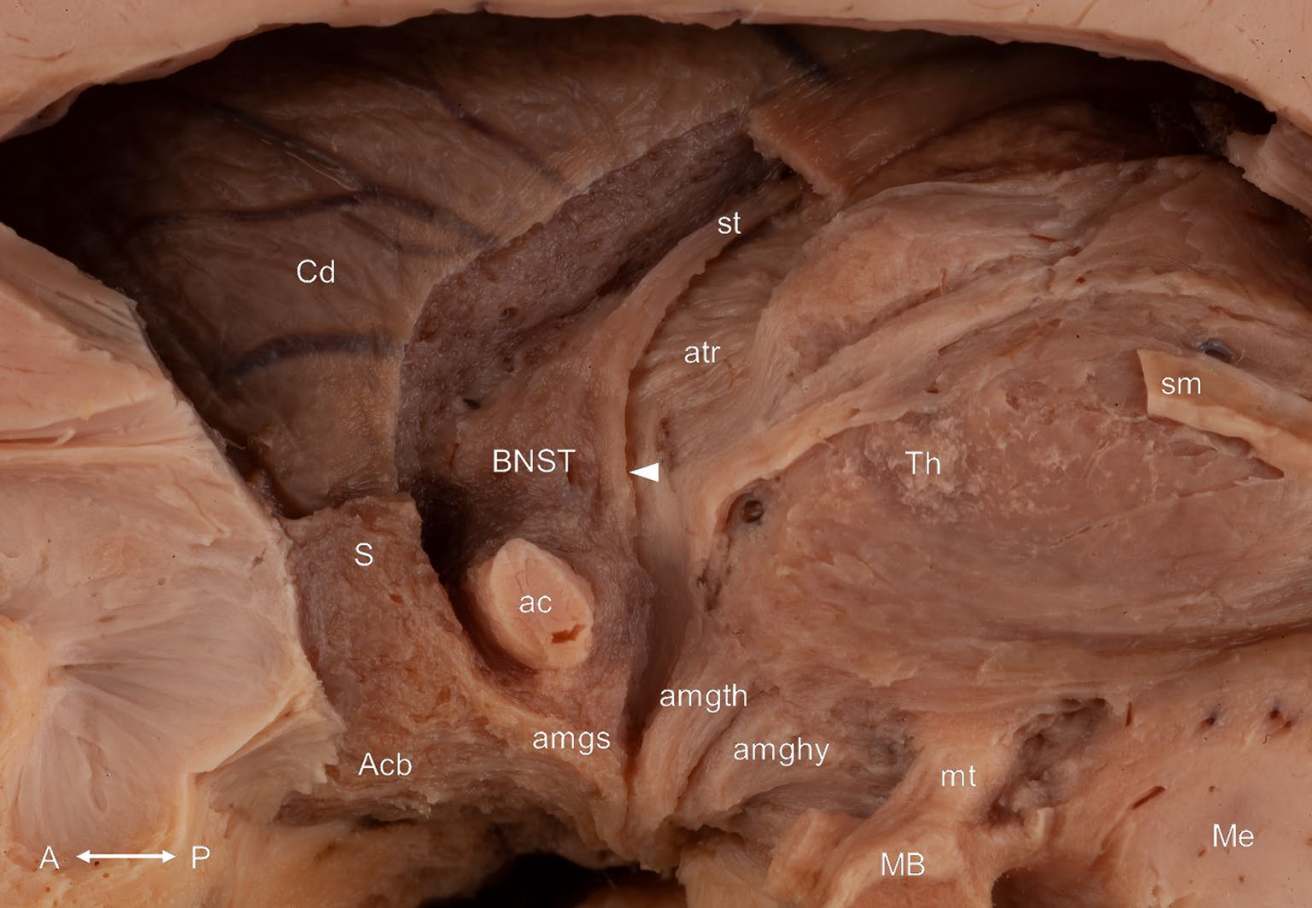
Overview of the deeper layer from a medial point of view. White arrowhead indicates the nuclear component of the stria terminalis. Acb: accumbens nucleus; ac: anterior commissure; amghy: amygdalohypothalamic fibers; amgs: amygdaloseptal fibers; amgth: amygdalothalamic fibers; atr: anterior thalamic radiation; BNST: bed nucleus of the stria terminalis; Cd: caudate nucleus; MB: mamillary body; Me: mesencephalon; mt: mamillothalamic tract; S: septal nuclei; sm: stria medullaris of the thalamus; st: stria terminalis; Th: thalamus. Compass: A: anterior; P: posterior.

**Figure 8:**
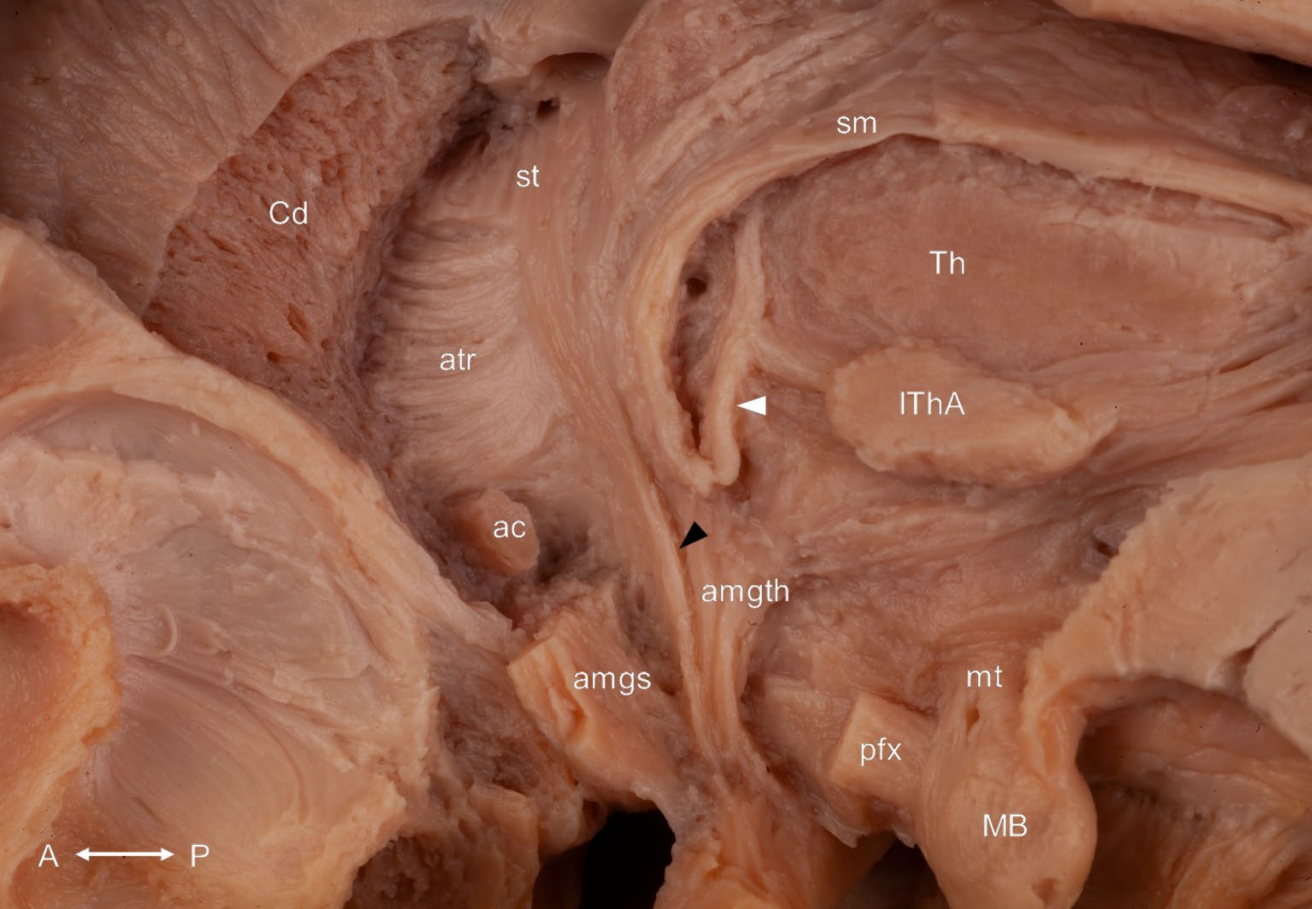
After the removal of the bed nucleus of the stria terminalis, the postcommissural component of the stria terminalis coursing to the hypothalamus was revealed (black arrowhead). The fiber bundle between the stria medullaris of the thalamus and the anterior thalamic nuclei can also be observed (white arrowhead). ac: anterior commissure; amgs: amygdaloseptal fibers; amgth: amygdalothalamic fibers; atr: anterior thalamic radiation; Cd: caudate nucleus; IThA: interthalamic adhesion; MB: mamillary body; mt: mamillothalamic tract; pfx: postcommissural fornix; sm: stria medullaris of the thalamus; st: stria terminalis; Th: thalamus. Compass: A: anterior; P: posterior.

The stria terminalis coursed in the sulcus between the caudate nucleus and the thalamus in the inferolateral corner of the pars centralis of the lateral ventricle and connected the amygdala with various regions. The careful removing most of the components of the septum and the postcommissural fornix revealed the distal part of this bundle. The terminating fibers of the stria terminalis could be distinguished in nuclear, precommissural and postcommissural parts. Most of its fibers terminated in the bed nucleus of the stria terminalis, a triangular-shaped grey matter structure located directly lateral to the septum verum and just dorsal to the anterior commissure. The removing of this nucleus revealed the postcommissural fibers reaching the anterior hypothalamic area. Some of these fibers intermingled with the fibers of the postcommissural fornix and the proximal fibers of the stria medullaris of the thalamus. The precommissural portion of the stria terminalis coursed just anterior to the bed nucleus of the stria terminalis and terminated on the medial side of the accumbens nucleus, rostral to the anterior commissure (Figs. 7, 8).

### Microscopic results

The histological analysis of the samples removed during the macroscopic examinations confirmed the correctness of the dissected fiber orientations. Fibers connecting the postcommissural column of the fornix to the stria medullaris of the thalamus (7/7; 100%), crossing fibers of the stria medullaris of the thalamus through the interthalamic adhesions (2/2; 100%) and presence of the precommissural fibers of the stria terminalis (3/3; 100%) were verified in every cases.

**Figure 9:**
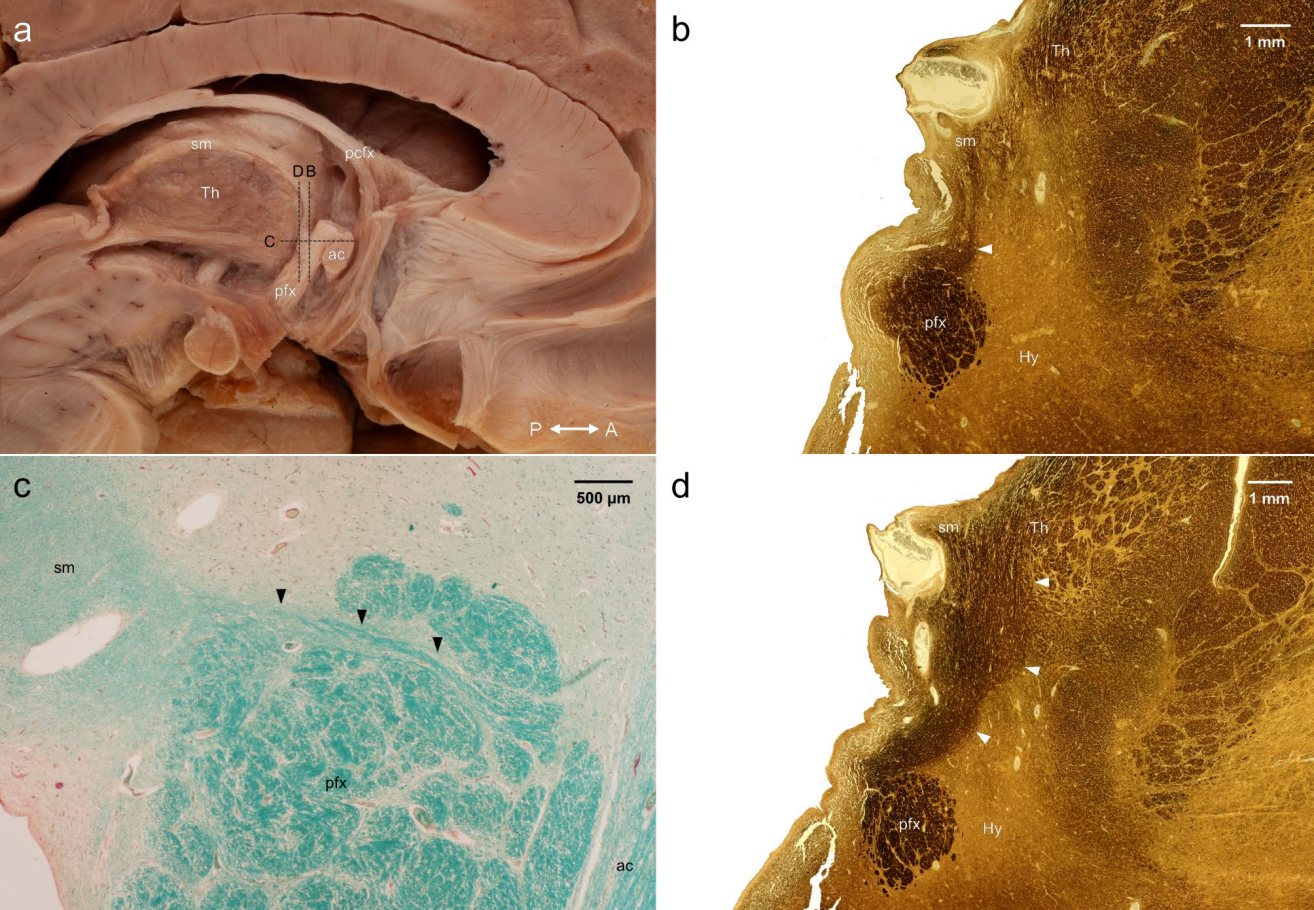
Histological validation of the fiber connections between the postcommissural column of the fornix and the stria medullaris of the thalamus as well as the anterior thalamic nuclei. The plane and localization of the histological sections are marked with a dashed line on Panel (**a**). Panel (**b**): coronal section showing the connection between the postcommissural fornix and the stria medullaris of the thalamus (white arrowhead). Silver impregnation, original magnification ×5. Panel (**c**): horizontal section at the level of the anterior commissure showing the connecting fibers between the postcommissural fornix and the stria medullaris of the thalamus (black arrowheads). Luxol fast blue combined with Sirius red, original magnification ×20. Panel (**d**): a few mm posterior to the plane of Panel (**b**), a large number of fibers can be seen leaving the postcommissural fornix and terminating in the stria medullaris and also in the thalamus (white arrowheads). Silver impregnation, original magnification ×5. ac: anterior commissure; Hy: hypothalamus; pcfx: precommissural fornix; pfx: postcommissural fornix; sm: stria medullaris of the thalamus; Th: thalamus.

The serial sections of the samples removed from the unfrozen hemispheres proved the presence of fibers between the column of the fornix and the stria medullaris of the thalamus (Fig. 9).

## Discussion

According to the description of Andy and Stephan, most of the septal nuclei found in animals are well developed also in humans, only the caudal group is mostly absent or at least significantly regressed^1^. The main difference in the human brain compared to the septal region of lower animals is the elongation of the septal nuclei in the vertical direction due to the appearance of the septum pellucidum. Andy and Stephan distinguished two parts of the human septum, the septum pellucidum and the septum verum^1^. Based on our results, however, these two parts cannot be sharply demarcated, since a part of the septal nuclei is located in the septum pellucidum, and the precommissural fornix, which is one of the most robust connections of the septum verum, also courses through it^13^. Horvath and Palkovits obtained similar results to those of Andy and Stephan regarding the structure of the human septum, but they used a simplified division, separating the medial and lateral nuclear groups^14^. They also distinguished the nucleus triangularis described as underdeveloped by Andy and Stephan, although they also noted that compared to the size of the whole septum, it is smaller in humans than in rats. Another relevant difference is that the bed nucleus of the stria terminalis belonging to the caudal nuclear group according to Andy and Stephan was no longer classified as a septal nucleus by them^14^.

The white matter connections of the septum verum in humans have already been depicted in a few diffusion-weighted magnetic resonance imaging (MRI)-as well as fiber dissection studies, however, the results of them are often inconsistent and none of them has yet focused on the topographical anatomy of this region. The stria medullaris of the thalamus was first visualized by Kochanski et al.^15^ and later by Roddy et al.^16^ using tractography. However, they did not describe the connection we dissected between the stria medullaris of the thalamus and the postcommissural fornix nor the crossing fibers of the stria medullaris of the thalamus through the interthalamic adhesion. Because of their small size and close relationship with the ventricular system, the visualization of these fiber streams likely exceeds the technical limitations of the tractography^17^. Moreover, these connections have not been reported in any previous fiber dissection studies. Kwon et al. depicted the stria terminalis for the first time with tractography, but they could visualize only its hypothalamic (postcommissural) component^18^. Later, Kamali et al. successfully depicted the pre- and postcommissural parts of the stria terminalis with high-resolution tractography. They also described an extension of the precommissural fornix to the medial temporal lobe^19^, however, we could not distinguish these fibers during our dissections. Li et al. also aimed the visualization of the amygdalofugal pathway by tractography. They could find only the nuclear component of the stria terminalis, but successfully depicted the amygdaloseptal fibers^20^. Our dissections could reveal all the subcomponents of the stria terminalis described by Klingler and Gloor^6^, except the commissural component. However, they also found these fibers only occasionally in their material^6^. Based on our dissections, the inferior fascicle of the septum pellucidum along with a small bundle of the cingulum also originates in the septum verum. The fasciculi of the septum pellucidum were first visualized using track-density imaging by Cho et al.^21^, however, to date only the fasciculus superior was further investigated by them, and they concluded that it is most likely a connection between the fornix and the prefrontal cortex^22^. Based on our current and previous^13^ results, the inferior fascicle of the septum pellucidum approaches, probably also intermingles with the fibers of the fasciculus superior, turns laterally then joins the fibers of the corpus callosum and presumably—similar to the fasciculus superior—travels to the prefrontal cortex via the forceps minor. Thus, we hypothesize that the inferior fascicle of the septum pellucidum may be a connection between the septum verum and the prefrontal cortex, but further studies are needed to confirm this assumption.

Our knowledge about the physiological and pathological functioning of the septum is still very incomplete and we can only infer it mainly based on animal experiments. Its role in the reward system was quite early revealed^23,24^. Currently, most of the information we have is related to the medial septum, which along with the diagonal band of Broca and the nucleus basalis of Meynert forms the most important cholinergic center of the brain^25^. Dysfunction of these areas for any reason, which may be damage or insufficient circulation of the anterior communicating artery or due to neurodegenerative processes, leads to memory disorders (basal forebrain impairment)^26^. One of the most significant relationships of the septal region is the bidirectional septo-hippocampal connection, which is carried out primarily via the precommissural fornix^27^. Through this pathway, the medial septum is not only involved in the cholinergic neuromodulation of the hippocampus, but also plays an important role in the rhythm generation and synchronization of hippocampal theta oscillations, which are also essential for the formation of memory traces^26^. In addition to its memory functions, the theta rhythm generation of the medial septum-diagonal band complex also plays an important role in the motivation of movement (locomotion) initiation^28^. We have much less knowledge about the function of the lateral septum. According to a recent review of Patel, it is primarily involved in processes related to anxiety and it mediates both anxiogenic and anxiolytic effects^29^.

The role of the septum in some pathological conditions was already proved also in humans. Using MRI, Butler et al. found enlarged volume of the septal area in patients with temporal lobe epilepsy without mesial temporal sclerosis^30^ before the clinical manifestation of Alzheimer’s disease^31^ as well as in healthy volunteers with increased accuracy of the source memory^32^. Brisch et al. found reduced neuronal density of the lateral septum but without accompanying volume changes in schizophrenic patients^33^. Heath et al. registered abnormal electrical activity of the septal area in patients with epilepsy and schizophrenia using deep brain electrodes^24^. Although the isolated lesions of the septum verum are very rare, they also suggest the complex function of this area. According to a case report, hypersexuality occurring after insertion of a ventriculoperitoneal shunt was experienced in two elderly men, where imaging showed the tip of the catheter in the medial septum in both cases^34^. Tumors affecting this region are also rarity and in most cases—due to the proximity of the ventricular system and the foramen of Monro—they primarily cause a disturbance in the circulation of the cerebrospinal fluid. However, beside increased intracranial pressure, memory loss, seizures and behavioral disorders have also been reported in the case of tumors localized in the septal area^35,36^. In everyday clinical practice, the ischemic lesions are the most important cause of damage to this region and its connections. Occlusion of the subcallosal branches of the anterior communicating artery supplying the septal area is clinically manifested by a sudden onset anterograde amnesia and confusion (acute amnestic syndrome)^37,38^. In addition to microangiopathic ischemic stroke, these tiny branches can also be damaged by surgical or intravascular treatment of aneurysms located in this region^37–40^. Isolated infarction of this artery was also reported due to local vasospasm occurring after subarachnoid hemorrhage caused by a ruptured aneurysm^41^.

In addition to its pathological changes, the clinical relevance of the knowledge of the septal region and its connections is also given by their potential use as a DBS target. The septum is one of the experimentally earliest stimulated brain areas. Olds and Milner's self-stimulation experiment of rats highlighted the role of the septum in the reward system^23^ which was later proven in human subjects as well^24^. However, it has now become clear that the septum has a role in much more complex functions, thus it may be a possible DBS target in several pathologies, primarily those related to the disturbance of hippocampal theta oscillations^2^. Jeong et al. demonstrated in a rat dementia model (basal forebrain cholinergic lesion) that hippocampal cholinergic activity and neurogenesis increased as a result of DBS of the medial septum and the rats' performance became as good as that of the control animals^42^. The medial septum may therefore be a promising target in Alzheimer's disease or Parkinson's/Lewy body dementia^26^ as well as in the case of cognitive dysfunction resulting from traumatic brain injury^4^. Based on animal experiments, stimulation of this area may also be effective in therapy-resistant temporal lobe epilepsy^3,43^. Some region in the vicinity of the septal region are already used as DBS target in the treatment of various psychiatric and cognitive disorders. Bilateral stimulation of the fornix as well as the nucleus basalis of Meynert is an experimental treatment in Alzheimer’s disease^44^. However, the results of these clinical trials are rather contradictory, which can be caused by the different locations of the electrodes within the fornix between the studies^44,45^. Since the precommissural fornix contains mainly cholinergic septo-hippocampal fibers, the isolated stimulation of this part of the fornix within the septal region might give more beneficial results. The subgenual cingulate cortex is also an intensively studied region as a DBS target mainly in major depression but also in obsessive–compulsive-disorder and schizophrenia^44,46^. The results of Howell et al. suggest that during stimulation of this area, the cingulum and the forceps minor are the most important connections that are activated and may be responsible for the therapeutic effect^47^. Our dissections showed that a bundle of the cingulum originated in the septal nuclei along with the inferior fascicle of the septum pellucidum, the fibers of which joined the corpus callosum and may reach the prefrontal cortex through the forceps minor. Although the modern definition of the septum handles it a separate entity, it should be mentioned that the bed nucleus of the stria terminalis is also an emerging target of DBS in the treatment of various psychiatric diseases, such as obsessive–compulsive-disorder, anorexia nervosa, addiction^44^ and major depression^48^.

During our fiber dissections, a less-known connection between the postcommissural column of the fornix and the stria medullaris of the thalamus was revealed, which was confirmed also by our histological validation. A macroscopic representation of this connection could only be found in the atlas of Pernkopf, in which it was designated as the hippocampo-habenular tract^49^. In addition to this name, it can also be found in the literature as the (medial) cortico-habenular tract, but most often it was simply referred to as the fibers connecting the postcommissural fornix to the stria medullaris of the thalamus. All information available about this structure is based on histological observations. First Gudden mentioned in his comparative study a fiber bundle leaving the fornix (“lateral uncrossed bundle of the fornix”), which, according to him, is connected to the stratum zonale of the thalamus and degenerates when the ipsilateral Ammon's horn is removed. Based on his observations, however, the stria medullaris of the thalamus only crosses the column of the fornix, without fiber exchange between them^50^. Later on, many authors confirmed or rejected the existence or the connection of this fiber bundle with the stria medullaris of the thalamus in several animal species. In the human brain, Marburg described the hippocampo-habenular tract undoubtedly in his study of the human habenula using histology^51^. In addition, the quantitative study of Powell et al. showed that only approximately half of the fibers coursing in the initial part of the postcommissural fornix reaches the mamillary bodies in humans, thus a significant number of fibers must leave it between these two points. They observed that this fiber loss is most gradual in humans, with fibers leaving the fornix along its entire length through the hypothalamus. According to their opinion, the fiber bundle originally described as the medial cortico-habenular tract consists of hippocampo-thalamic and septo-thalamic fibers^52^. Based on the literature, the existence and origin of this connection as well as its termination are very contradictory, and it is very likely that there are also interspecies differences. It is assumable that this bundle may contain fibers of both septal and hippocampal origin. Our results unambiguously confirmed the connection between the postcommissural column of the fornix and the stria medullaris of the thalamus, however, due to the nature of the fiber dissection technique, we could not determine the origin and termination of these fibers. It is possible that they reach the habenula or they only temporarily join the stria medullaris of the thalamus and terminate in one or more of the thalamic nuclei. So far, only a few data in the literature suggests some clues about the character of this connection. Watanabe et al. showed in their study that during brain development, the cells of the nucleus triangularis and the bed nucleus of anterior commissure belonging to the caudal septal nuclear group originate from the anterior part of the diencephalon (eminentia thalamica) in rats. These cells develop directly behind the interventricular foramen and migrate through the fornix in a rostrodorsal direction to their final location in the septum, while their axons contact the medial habenula via the stria medullaris of the thalamus^53^. This could explain why some septal fibers may follow this route. Goutagny et al. showed in their electrophysiological study of rats that although we do not know a direct anatomical connection between these two structures, there is a functional connection between the lateral habenula and the hippocampus. Moreover, the lateral habenula influences hippocampal theta oscillations and plays a role in memory functions^54^. The fiber bundle dissected in our study can represent this connection either directly or through the septal region. The injury of these fibers could be a possible complication of surgical procedures in the anterior part of the third ventricle through the interventricular foramen due to their close anatomical relationship. The exact function and possible clinical significance of this structure needs to be clarified in the future.

### Limitations and further aspects

Due to their size, several structures described in our study are at the limit of the fiber dissection technique. Their strong relationship with the ventricular ependyma made their dissections even more challenging. In some cases, we were uncertain about the accuracy of the obtained results, therefore, we performed histological validation by taking samples from the dissected areas, which confirmed the correctness of the dissected fiber orientations in all cases. Certain connections of the septum described in the literature, such as the septo-hypothalamic fibers or between the single septal nuclei, cannot be examined with this method. On the other hand, we were able to follow many small fiber bundles that could not been described using tractography so far. Considering the clinical relevance of it, further human studies are needed to obtain more information about this brain region.

## Data Availability

All data generated or analyzed during this study are included in this published article.
